# Industrial Ultrasound Applications in the Extra-Virgin Olive Oil Extraction Process: History, Approaches, and Key Questions

**DOI:** 10.3390/foods8040121

**Published:** 2019-04-12

**Authors:** Maria Lisa Clodoveo

**Affiliations:** Interdisciplinary Department of Medicine University of Bari-Piazza Giulio Cesare, 11-70124 Bari, Italy; marialisa.clodoveo@uniba.it; Tel.: +39-334-605-3-605

**Keywords:** olive oil, extraction process, innovative approaches, ultrasound, technological level of readiness

## Abstract

Taking an idea from a basic concept to a commercially available product is highly rewarding, but it can be a very long, complex, and difficult journey. Recognizing and understanding the stages of the process and using the right support to help you navigate through it can mean all the difference between success and failure. The road from concept to market is marred with obstacles, and many businesses fail to pass beyond the development stage. A better understanding of the innovation process is essential from the outset if the pioneers of innovation are to overcome the dangers that they are likely to face along the way and maximize their opportunities for success. In the olive oil sector, the most recent radical innovation is the introduction of ultrasound into the industrial extraction process. Many efforts have been made in order to overcome the Valley of Death. The strategy of designing, implementing, and testing an innovative system that combines the mechanical energy of ultrasound with the possibility of modulating the thermal exchange of olive paste (heating or cooling) has enabled the following: (1) Eliminating malaxation by realizing a real continuous process; (2) raising extraction yields by recovering a further quota of extra-virgin olive oil that is usually lost in the pomace; (3) improving the content of antioxidant molecules simultaneously with yields; and (4) offering a sustainable plant solution that can guarantee the right income for producers.

## 1. The Olive Oil Sector and the Evolution of the Oil Miller Profession

The growth of the virgin olive oil sector is linked to the development of its innovative capacity. Understanding the factors that can determine the potential for innovation means understanding the main levers of intervention for the competitiveness and growth of an entrepreneurial activity threatened by an evolving global context, which leads traditional producer countries to lose positions of prestige that they always had in the international context.

In the last 50 years, the olive oil sector has undergone a process of transformation not dissimilar to other productive contexts. Thanks to an increase in the level of education and training of olive millers, from agricultural and food science to economy and marketing, coupled with technological improvements in production processes, the key roles of knowledge and human capital in determining the competitive growth of the olive oil sector are increasingly clear. Clarifying the value of skill creation, the technology progress, and continuous learning in determining the performance of an olive mill is a key point in promoting "knowledge" as the primary engine of productivity and growth of the entire olive oil sector.

The food industry is, in general, characterized by permanent innovation processes that require continuous technological, commercial, and bureaucratic legal updating [1]. In line with this trend, the profession of olive miller has seen its field of competence and action expand in relation to the entire olive oil production chain [2]. The modern olive miller is able to get the best oil possible, considering the quality of the raw material, within a specific territory, respecting a tradition that evolves and changes and above all looks toward new trends in the market [3].

The modern olive miller has to understand the potential of technology and has to be able to contextualize it in the geographical area in which he operates. Extra-virgin olive oil is a well-defined project of an olive miller, and it is the result of interactions between machines that constitute an industrial plant and raw material characteristics. This result should never be a surprise. This is because from olive orchard management to the bottling phase, there are hundreds of decisions to be made consciously. It is important to know the consequences of these decisions. At each crossroads, it is necessary to know which is the road that moves away or approaches the initial project. The olive oil project, of each olive miller, has to be a marketing project tailored to a specific target market to which the olive miller has decided to direct production and sales. In fact, during all operations, ranging from crushing, to malaxation and to the centrifugal separation [4,5,6,7], complex phenomena of a physical, chemical, and biochemical nature are established. These simultaneous reactions make virgin olive oils different from other vegetable lipid matrices through their composition, sensory, and health properties, thanks to a transfer from olive olive paste to the oil of minor compounds (both constitutive of the fruit) and neoformation [8]. In fact, it is possible to modulate, by activation or inhibition, these complex phenomena of a physical, chemical, and biochemical nature occurring on a microscopic scale through the control of operating macroscopic parameters such as specific energy (J/kg of olive paste), temperature (°C), processing time (s), and composition of the atmosphere in contact with the olive paste ([O_2_]) [9,10,11,12].

A profound change in the paradigm of a profession that has existed for millennia is occurring for the modern olive miller based on his new degree of competence and his recent awareness relative to the possibility of modulating the production process as a function of a predetermined quantum–qualitative project. The miller of the past was relegated to the role of mute spectator who superintended a process whose outcomes were often unknown to him (for the Latins, an *opifex* or *operarius*, i.e., a worker assigned to manual labor) and who was not able to build solid business strategies based on segmentation, targeting, and product positioning.

The contemporary miller, thanks to the technological progress of machines and acquired scientific knowledge, has become an *artifex*, an artificer, or one who exercises an *ars* (art in Latin), which is intended as an activity that requires complex technical knowledge at the service of a particular attitude. In conclusion, an artifex is an olive miller able to use machines in order to "modulate" the enzymatic activities that take place in the olive paste, consciously modifying the chemical, organoleptic, and health characteristics of the resulting virgin oil. Modular is a term borrowed from the world of music. In the musical field, this verb indicates the ability to vary a sound or a tone in order to obtain a harmonious effect. It is, therefore, an indispensable term in describing the activities of the contemporary olive miller. In a very short period, he has to make a series of decisive choices to obtain extra-virgin olive oil with an overall pleasant sensation, due to the perception of its components, phenolic and volatile compounds, which act as olfactory–gustatory, tactile, and kinesthetic stimuli perfectly balanced between them.

This evolution could be compromised by the limits of the operating conditions of the machines that compose the oil mill. In order to overcome the existing gaps that still compromise the effectiveness and efficiency of the process, more flexible and adaptable solution must to be developed though a trans-disciplinary approach (Table 1) [13]. "Effectiveness" and "efficiency" are two words often used as synonyms, but which actually reflect two distinct concepts. Effectiveness, in fact, indicates an ability to achieve a set objective, while efficiency indicates the ability to do it using the minimum necessary resources. In the case of olive plant innovation, technology is effective if it is able to increase virgin olive oil yields and antioxidant content by reducing their losses in byproducts, and it is efficient if it achieves these goals in a sustainable manner, reducing energy costs in terms of benefits for business economies and the impact on the environment [14].

## 2. The Role of Research in Innovation: History and Approaches Relative to the Implementation of Ultrasound in the Virgin Olive Oil Extraction Process

Innovation and scientific research, together with technology transfers, are crucial factors in facing the challenges of the future. Moreover, they are essential elements for a sustainable development model that is able to determine economic growth suitable to meet the needs of the international olive oil system in terms of well-being in the short, medium, and long terms, responding to the needs of the present without compromising the expectations of future generations.

A needs analysis is the starting point and the essential step in determining the objectives that the innovation process has to pursue.

From an ideal perspective, the development model to be pursued in a modern vision of the olive oil sector [15] should simultaneously attainseveral objectives:

- Guaranteeing greater productivity;
- Raising the quality of production;
- Intercepting the growing demand for foods with recognized healthy actions characterized by a premium price;
- Strengthening identity and the multiplicity of product offerings through the characterization and valorization of sensory profiles;
- Segmenting the offer by introducing systems for the identification of high-end products; and
- Guaranteeing a fair income and a fair distribution of value along the supply chain, also through the exploitation of byproducts [16].

In this direction and in response to innovation needs emerging from a demand–pull approach, research lines focused on the application of ultrasound combined with heat exchange in the extraction process of extra-virgin olive oil have been developed.

The idea of experimenting ultrasound [17] in the extraction process of extra-virgin olive oil first arose from a dual need:

- On the one hand, to develop a plant solution that represented a radical innovation capable of eliminating the malaxing phase [18]; and
- On the other hand, to recover a percentage of virgin olive oil (about 3%) that, due to the technology currently available on the market, is dispersed in the pomace, fueling the market of vegetable oil with lower nutritional and economic value (pomace olive oil), which is a low-cost substitute product of extra-virgin olive oil and therefore an internal competitor in the supply chain [19].

Both of these aspects, if resolved, would have immediate and positive effects on the entire international olive oil sector [20,21,22,23,24].

Starting from the necessity of plant-type innovation, it should be remembered that the malaxation phase is currently considered a so-called "necessary evil" in optimizing the centrifugal separation of the oil inside the decanter [6]. In fact, crushing, the first stage of the virgin olive oil continuous extraction process, determines the rupture of the drupe into coarse fragments containing hundreds of cells that pass intact through the mechanical device. The cellular breakage is not pushed further in consideration of two factors negatively linked to a possible surplus of mechanical energy:

- The increase in temperature of the paste, a factor that would compromise the quality of the resulting oil; and
- The risk of emulsion, an element that could damage oil extraction yields.

Malaxation is therefore considered a phase in which many transformations of a mechanical, physical, chemical, and biochemical nature, both desired and undesired, take place simultaneously during a long period of time not suitable for rigorous and reproducible control conditions (also due to the convulsive work rhythms related to the brevity and intensity of the olive oil harvesting season). Long malaxing times, as well as being a threat to the quality of oil, make this phase of mixing of the olive paste at a controlled temperature the "bottleneck" of the continuous process [25].

From an engineering point of view, bottleneck is a phenomenon that occurs when the performance of a system or its capabilities is strongly constrained by a single component. Bottlenecks occur when the incoming workload reaches the machine at a faster rate than it can handle, thus limiting the overall speed of the entire process. Having a bottleneck in the process therefore tends to create a queue and increase the overall cycle time. Bottlenecks cause stalls and slowdowns in the production flow. Therefore, with equal resources, production is slower, and fewer quantities are realized respect to the optimum process. Efficient bottleneck management in the olive oil extraction phase can lead to huge benefits in terms of the effectiveness (more oil yields, more phenol compounds, and a pleasant and harmonic sensory profile) and efficiency of the process (less time, fewer costs, less energy).

In an olive mill, at present, the limited working capacity of a malaxer penalizes the production efficiency of the decanter: The main system solution adopted to manage this inefficiency is to multiply the number of malaxers, either in series or in parallel, to ensure the continuity of the process, which is not without an increase in investment costs for the olive miller.

The process line of an olive mill can be compared to a chain, whose strength depends on its weakest link: Malaxation. If the goal of the innovation process is to make the chain stronger, improving production, a reinforcement of this weak link is needed, defining the objectives of the phase and introducing an effective and efficient innovation that achieves the same goals by limiting inputs (time, energy, water, losses of oil and polyphenols in byproducts).

A strengthening of the weak ring must therefore be such that the performance of innovation is superior to the phase to be innovated. Malaxation is the step that quantitatively and qualitatively modulates virgin olive oil production.

It is well known that the quality of oil and oil yield are parameters in antithesis, and therefore every operating choice carried out with the machines currently present in the olive mill allows the olive miller to choose as a target predominantly the quantity or predominantly the quality of the product. Therefore, he can obtain a large quantity of standard virgin olive oil (low polyphenol content) or a small quantity of high-quality virgin olive oil (high polyphenol content).

Moreover, many operators ignore that malaxation is, first of all, a finishing of the crushing phase due to the cutting action of the fragments of pits during the mixing that tear the cells passed intact to the oil mill (in a delicate way but for an extremely long time) [26,27,28].

In summarizing the desired effects from an introduction of a new machine, it is necessary to develop a process that is able to determine a delicate break of the cells passed intact through the crusher and simultaneously to do the following:

- Avoid emulsions and undesired temperature rises [3];
- Accelerate the coalescence phenomena of the minute oil droplets freed from elaioplasts (leukoplasts specialized in storing lipids) [15];
- Allow the dissolution of biophenols from the aqueous fraction of the olive paste toward the oily phase [14];
- Favor the enzymatic synthesis of the pleasant volatile compounds [3]; and
- Limit the oxidation reactions of the load of fatty acids and polyphenols [14] in a system that effectively operates continuously by transferring the olive paste directly from the olive crusher to the decanter without penalizing the working capacity of the latter.

It is clear that the levers traditionally used to improve the efficiency of the malaxing phase (improvement of the ratio volume/surface of the malaxer, temperature increase, water addition, etc.) cannot be stressed beyond the boundaries already achieved. Moreover, these conditions, if they are useful in improving oil yields, on the other hand can compromise the quality of the product [6].

When in a certain industrial sector the available technology proves insufficient to improve process performance, it is necessary to look at the landscape of emerging technology. These are radically new technologies that are able to offer the possibility of opening new areas to scientific knowledge and technical applications. This is the case for ultrasound applied to the virgin olive oil sector.

Ultrasound is sound waves with frequencies higher than the upper audible limit of human hearing (greater than 20 kHz) [17,30]. The use of low-frequency ultrasound (20 kHz) is a strategy to reinforce the weak link in the process of continuous extraction of extra-virgin olive oil thanks to the mechanical effects that the sound waves induce within the olive paste. The ultrasound propagating in a liquid determines the alternation of positive and negative pressures inside it. When the negative pressure values are below the vapor pressure of the fluid itself, it undergoes a phase change from liquid to gas, forming cavities containing steam and giving rise to the phenomenon of cavitation. Cavitation is a physical phenomenon consisting of the formation of cold vapor bubbles inside a fluid that then implode, producing shock waves, i.e., pressure waves that can be extremely intense. If implosion occurs near the cell wall of the olive fruit (cells passed intact through the crusher), it generates a liquid microjet that breaks the wall, freeing the cell contents, all within a few microseconds. The mechanical effect of the acoustic cavitation breaking the intact olive cells frees further portions of oil and minor compounds. In addition, whirling motions impressed on the olive paste by the pressure transients determine the coalescence of the lipid droplets [29]. The introduction of ultrasounds into the olive oil can be considered a radical innovation because it delivers a creative radical solution to a challenging problem.

Thanks to a transdisciplinary approach, it has been possible to realize the first industrial-scale prototype (a sono-heat exchanger) able to eliminate virgin olive oil process bottlenecks and break the historical paradigm between yield and quality of virgin olive oil. In fact, a sono-heat exchanger placed between the crusher and the decanter is able to realize an effective and efficient continuous process, obtaining a simultaneous increment of both oil yield and quality.

Research innovations are constantly occurring in universities, research institutions, and industrial research laboratories. These are reported in the scientific literature and presented to the scientific community in various congresses and symposia. However, the conversion of these research results to industrially useful innovations is considerably more complex than generally appreciated.

The realization of the prototype that combined ultrasound for the first time with a heat exchange was the result of a planned technology development roadmap emerging from a research path. The path forward started from a lab-scale observation and finished with a full-scale device ready for the market that passed through all the research and development tasks needed to increase the levels of maturity of ultrasound applications in the virgin olive oil extraction process. Tasks have included modeling, testing, bench-scale demonstrations, pilot-scale demonstrations, and fully integrated prototype demonstrations.

**Figure 1:**
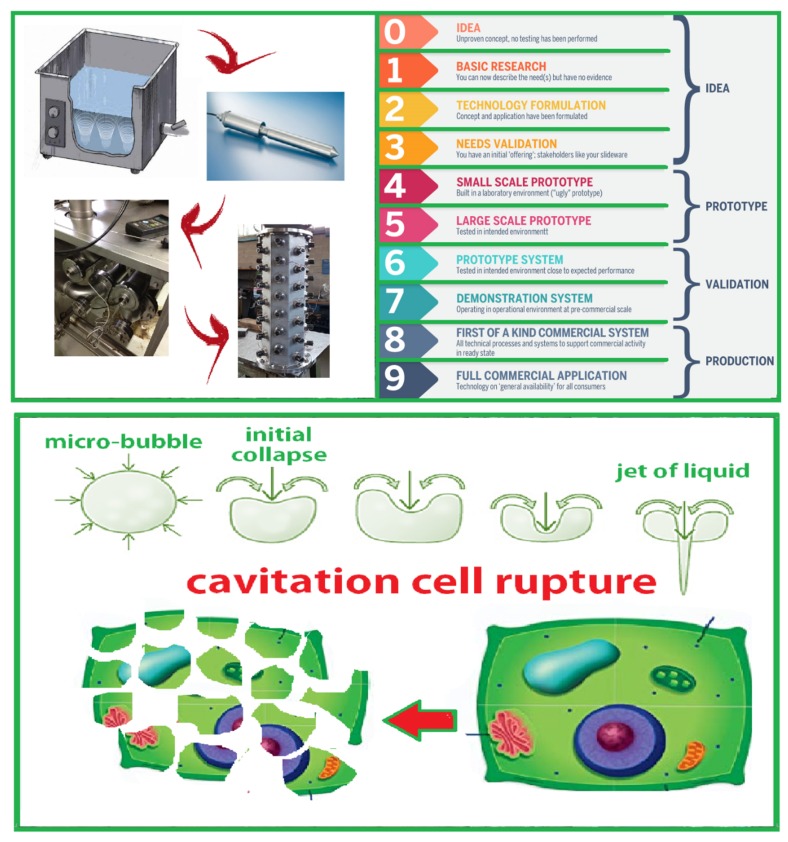
The long, complex, and difficult journey to pass from the basic concept of an idea to a commercially available product, the TRL (Technology Readiness Level) definitions, and the mechanisms of cell rupture due to cavitation phenomena.

In order to better describe the research and development strategies and the milestones that have marked the road from the first observations in the laboratory to the full-scale industrial plant, a technology maturity assessment system (TRL: Technology readiness assessment) is used. The TRL process originated with NASA (National Aeronautics and Space Administration) to evaluate the deployment readiness of a technology and its readiness to function in an integrated environment. A technology readiness (TRL) is assigned to each evaluated technology based on its relative level of development toward deployment. TRL definitions are shown in Figure 1.

The first paper (in 2011 [36]), an overview on the application of emerging techniques in the virgin olive oil extraction process, describes the strategies useful in developing innovative plants and demonstrated the transition from TRL 0 to TRL 2. This was a review that started from the development of an idea (TRL 0: Unproven concept, no testing has been performed) examining the principles postulated and observed without any experimental proof available (TRL 1: Basic research). The paper analyzed the potentials of a group of emerging technologies (ultrasound, microwaves, and pulsed electric fields) to improve the virgin olive oil extraction process and concluded the overview with the formulation of a hypothesis of application of ultrasound technology to the virgin olive oil extraction process (TRL 2: Technology formulation).

**Figure 2:**
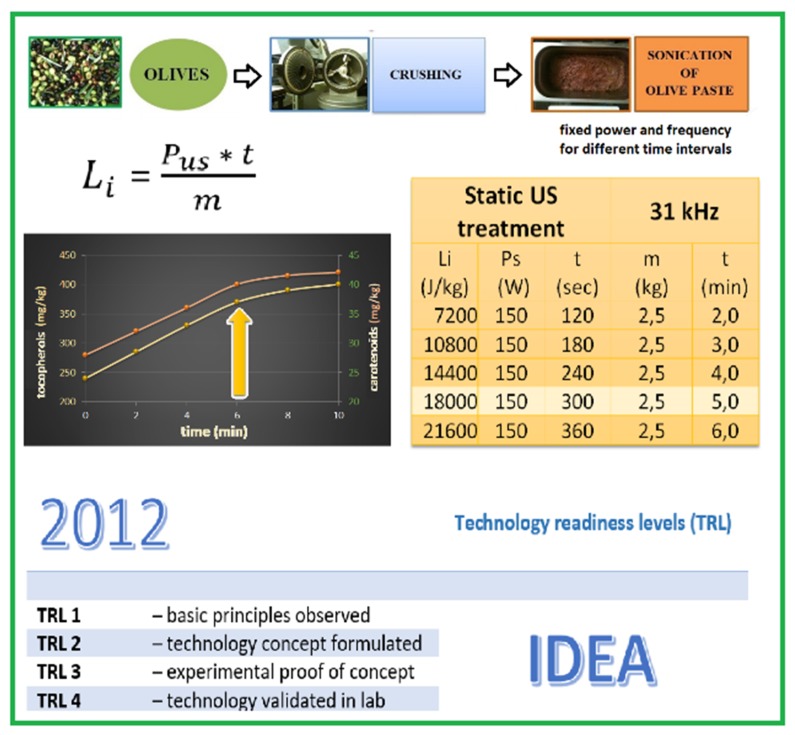
From the basic principles observed to the technology validated in a laboratory.

The first laboratory tests (Figure 2) (TRL 3: Applied research, proof of concept, and TRL 4: Small-scale prototype built in a laboratory environment) were conducted in 2012 [17,25,37]. The effects of ultrasound on the oil yields and on the minor compound contents were evaluated by means of an ultrasonic bath of two liters of capacity.

The ultrasonic bath was characterized by fixed frequency and power: Varying the timing of sonication, different amounts of specific energy (J/kg) were given to the olive paste.

The extremely encouraging results are summarized as follows:

- The ultrasonic technology, under the tested conditions (low frequency and high power), reduced the time of extraction of virgin olive oil;
- The ultrasound technology did not modify the chemical parameters used in the product classification of the product;
- Ultrasound technology did not cause the onset of sensory defects in the product;
- Ultrasound technology did not imply an increase in energy consumption;
- Ultrasound technology increased amounts of minor compounds with healthy effects;
- Ultrasound technology increased amounts of oil, reducing losses in byproducts.

The subsequent steps were the most complex and required the integration of disciplines that were distant from each other to establish a sudden acceleration of the development of technology.

The transdisciplinary approach has allowed us to face simultaneously the numerous issues that separate the idea from its transformation first into an invention (creating something new that the market has not seen before) and then into an innovation (taking an existing concept or idea and improving it) able to change working methods in the real world. The olive oil sector is extremely conservative from all points of view: Both of producers consumers, but also on the level of policymakers (relating to legislative frameworks and marketing standards).

The transdisciplinary approach allowed for exploring opportunities and threats simultaneously in order to verify how advantageous it was to implement a radical innovation (ultrasound) in a traditional sector (olive oil sector), reducing risks and overcoming obstacles exacerbated by the intrinsic characteristics of the oil supply chain. In fact, one of the major problems linked to the development of innovations in the oil sector is due to the fact that the harvesting season lasts only three months each year. Moreover, the climatic variability of different years [38,39,40,41,42] makes experimental conditions, which are linked to initial fruit quality, unrepeatable, and it becomes extremely complex to exploit the results of the observations in strategies of rapid optimization. This condition implies that it is necessary to introduce useful technologies to overcome the limits of the empirical approach.

The first obstacle to overcome certainly concerns the availability of economic resources to support TRL escalation. In a financing scenario, the public sector generally intervenes at the beginning of the innovation process, financing basic research (starting from the laboratory) that generally has not yet identified a specific end product and is characterized by a high degree of uncertainty. Two different public funds EAFRD (the European Agricultural Fund for Rural Development), with which the EU cofinances the RDP (Rural Development Program), and ESF (the European Social Fund) enabled the creation of the first and second industrial prototypes [38]. These funds allowed researchers to test different solutions in plant engineering, tackling the limits linked to the rheological complexity of the matrix (olive paste) and reaching the optimal geometry after many difficulties.

The first design step required a strategy that would combine a device already operating in the olive mill with the models of ultrasound equipment available on the market. The first approach then followed the philosophy of incremental innovation (a series of small improvements to an existing product). The ultrasound market made available two types of different devices: Probe-shaped transducers and plate-shaped transducers. The first dilemma to be solved was to understand which machine could be equipped with ultrasound to create a sustainable plant solution, both from an economic and an energetic point of view. The choice fell to a triple tube heat exchanger (already widely used in the market to shorten malaxing times) combined with ultrasonic probe-shaped transducers.

**Figure 3:**
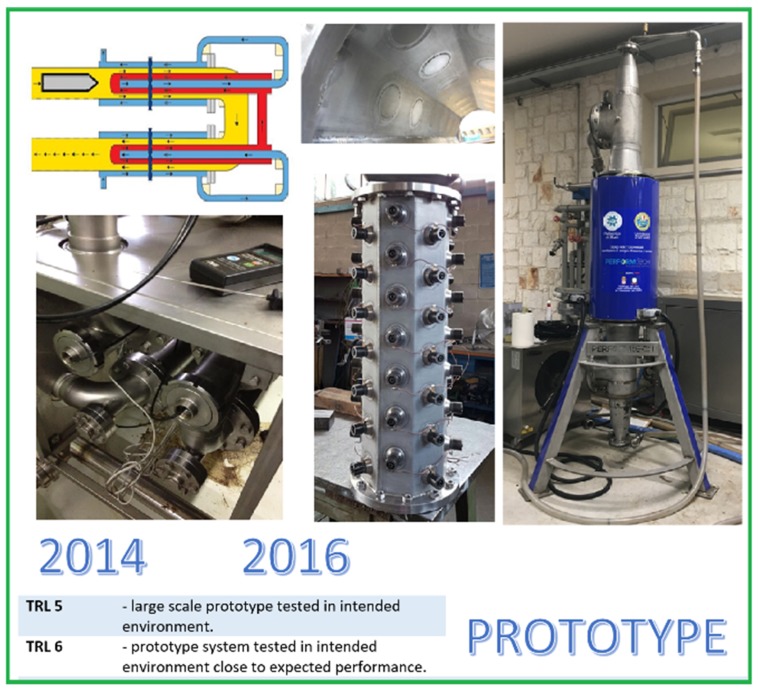
The industrial prototypes.

The machine just described is shown in Figure 3 and represents TRL 5 (a large-scale prototype tested in its intended environment). As physiologically expected from a research activity conducted in the intended environment, the experimental tests were useful in tracking the road to be pursued to overcome the application limits found.

During the harvesting season in 2014, the industrial application of ultrasound confirmed the laboratory observations, and the results of the first prototype application are summarized as follows:

- The ultrasound extraction of virgin olive oil was faster than traditional process;
- The amount of oil extracted by means of ultrasound increased by 10% (about 1.5 kg of extra oil per 100 kg of olives);
- The extracted oil by means of ultrasound maintained the chemical characteristics of the product classification unaltered;
- The quantity of polyphenolic molecules, carotenoids, and tocopherols increased as a result of the treatment of olive olive paste with ultrasound;
- The sensory profile of the oils extracted with ultrasound was more harmonic than the control;
- The energy commitment required by the use of ultrasound was modest.

The limits of the application of the first prototype were related to aspects of functionality. The combination of the ultrasound wave transducer with the geometry of the triple tube heat exchanger caused problems with the continuity of the flow of olive paste in contact with the probe. When the layer of olive paste in contact with the probe was not uniform (effect of the pump upstream of the heat exchanger), and pockets of air disaggregated the fluid, the propagation of the sound wave could not take place and the probe set itself automatically in protection mode and switched off.

During the harvesting season in 2015, the first prototype was modified by placing the ultrasonic probes in vertical rather than horizontal tubing. The question of the continuity of the olive paste was solved, but the operating pressure conditions in which the paste was fed into the heat exchanger were excessively drastic and could counteract the desired cavitation phenomenon.

At the end of the tests on the first prototype, it was possible to state the following:

- The industrial application of ultrasound in the virgin olive oil extraction process was possible, and it made the process efficient and effective;
- The combination of the ultrasound probe-shaped transducer with the triple-tube heat exchanger was not the best plant solution and required further design efforts to offer the olive miller a robust, reliable, and friendly machine.

Every obstacle in the experiment changed the research perspective, helped reframe the experimental design, and led to an increasingly refined approach to the problem, narrowing iteratively over time the possibilities for fruitful study.

The second design approach then followed the philosophy of radical innovation (an invention that wholly replaced an existing process to create something substantially new and unique).

In this phase, an empirical constructive approach based on a combination of a device already operating in the olive mill (triple tube heat exchanger) and an ultrasound probe-shaped transducer was integrated with a theoretical approach that led to a fully innovative system.

The principles from which the innovative design approach started can be summarized as follows:

- Constructive simplification for a reduction in costs;
- Numerous and independent low-power transducers (button-shaped transducers) instead of two high-power probes, so that in the case of partial failure, the machine continued to work without interrupting the process;
- Modularity of the section hosting the transducers for simple scalability of the technology;
- Flexibility in the heat exchange (heating and cooling to cope with the phenomenon of early harvesting and climate change); and
- The possibility of regulating temperature and ultrasonic energy in order to offer a tool to the olive miller to modulate the chemical, sensorial, and health quality of the product.

Before proceeding with the technical design of the innovative device, a numerical study combining a simulation of pressure transients induced by the sound wave through the olive paste with fluid dynamic phenomena was conducted.

This type of approach had distinct purposes. Considering that olive paste is a three-phase fluid composed of two liquids immiscible with each other and a solid component characterized by high viscosity, it was necessary to define the area of propagation of the ultrasound waves within which effective cavitation phenomena were realized.

An evaluation of the dimension of the effective propagation area of the sound wave allowed for establishing three different aspects of the constructive geometry:

- The thickness of the olive paste completely invested by the ultrasonic treatment;
- The minimum distance of the steel wall in front of the ultrasonic transducer to assure that cavitation, which is useful in breaking drupe cells, did not represent a risk of damage to the plant structures; and
- The minimum distance between two transducers to prevent overlapping of the effective sonication areas that could reciprocally neutralize effects or cause damage to the transducers.

The result of the simulation study was an innovative geometry for the sono-heat exchanger.

**Figure 4:**
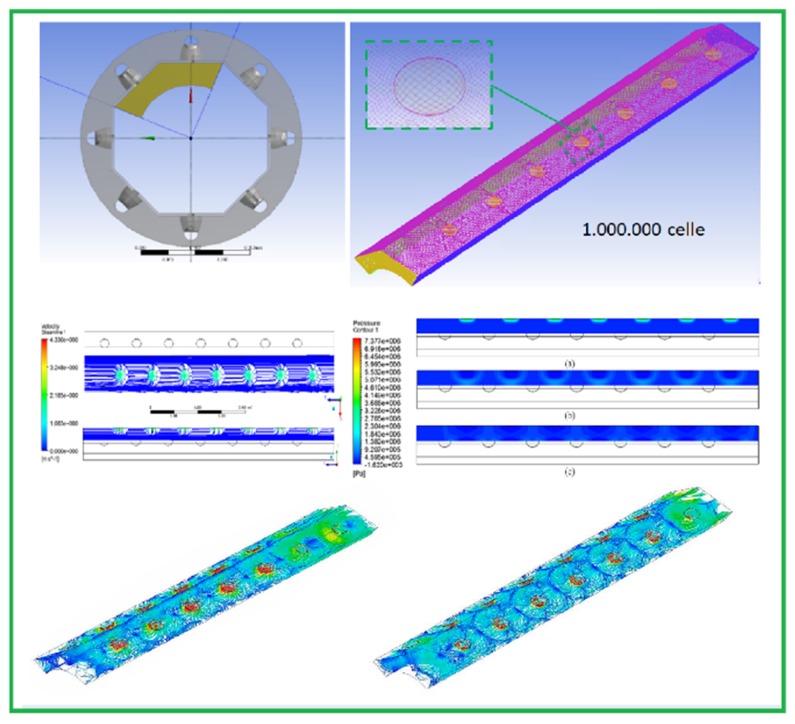
Results of the numerical simulation.

**Figure 5:**
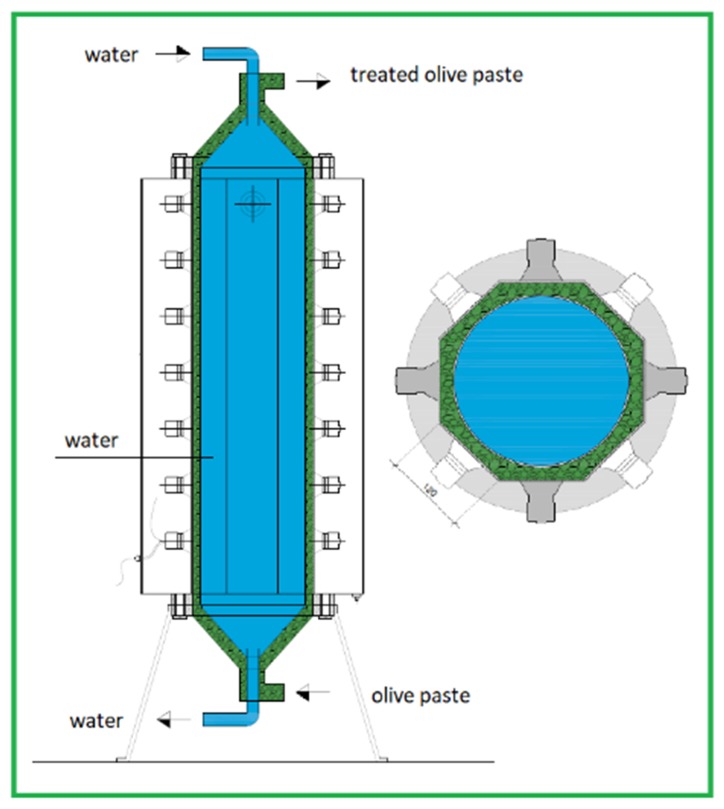
The innovative sono-heat exchanger equipped with button-shaped transducers.

The innovative sono-heat exchanger, employing button-shaped transducers, was composed of a couple of annular sections (Figure 4 and Figure 5).

Olive paste flowed into the external annular section, while water flowed into the internal annular section to control the temperature inside the olive paste. Outside the external annular flow section, a transducer for each side of the octagonal shape section was set up to provide ultrasounds. The energy per kilogram of olive paste was the result of the power of each transducer combined with the flow rate flowing inside the sono-heat exchanger.

During the harvesting season in 2016, the prototype system was tested in an industrial olive mill, transforming tens of tons of olives in oil of high quality and rich in polyphenols, with a concentration compatible with an application of the health claim of biophenols approved by the European Food Safety Authority (EFSA) and with the expected performance (TLR 6).

It is clear that in the process of technological innovation, it is necessary to work on two distinct levels:

- On the one hand, overcoming technical difficulties related to the scale-up of a technology; and
- On the other hand, needing to create and organize a complex system for the innovation that succeeds in involving numerous actors and stakeholders along a process articulated in phases ranging from discovery to full technological maturity.

Both of these fronts can bring innovation to the threshold of the so-called “Death Valley”. The main cause of loss of a technology in “Death Valley” is given by reduced financial resources and limited public support. In the financing scenario, the public sector does not intervene if the level of technological maturity is between TRL 6 and TRL 8, as it would constitute an intervention in favor of the private sector. On the other hand, for the private sector, this level of technological maturity, despite being tied to a potential profit, still has a high risk of commercial failure, and many technological obstacles can separate it from the market. “Death Valley” refers precisely to this intermediate moment of financial support between the public and private sectors. In this way, a promising technology may not progress along the demonstration and development phases necessary to achieve its full spread. In this condition the innovation cannot give a contribute to improve the competitiveness in the concerned sector. This is the point of no return from which many new ideas that go through the process of innovation cannot progress.

The technological innovation related to the use of ultrasound in the extraction of extra-virgin olive oil could overcome the threat of the “Death Valley” stall thanks to the financial support of the following:

- Instituto nutrizionale Carapelli (in order to overcome the logistical obstacles linked to the transfer and installation of the prototype in different olive mills); and
- The COMPETiTiVE project (Claims of Olive oil to iMProvE the market ValuE of the produce), financed by a group of foundations of a banking origin (AGER: Agroalimentare e ricerca), which was indispensable in the characterization of the matrices and the validation of the results.

**Figure 6:**
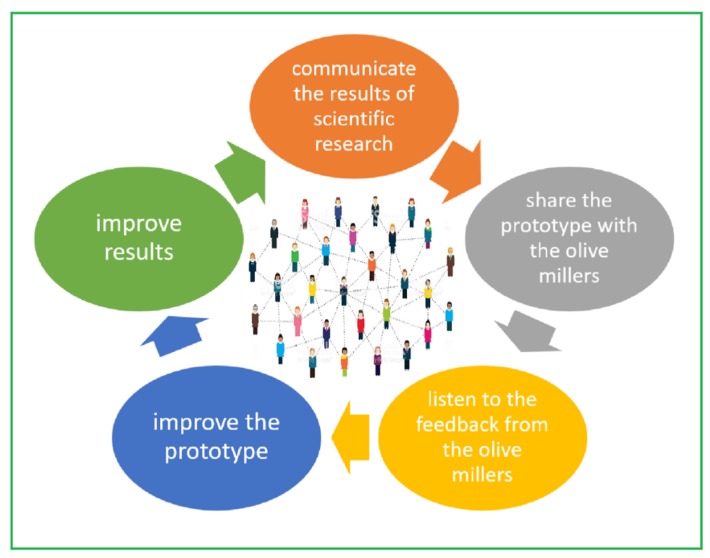

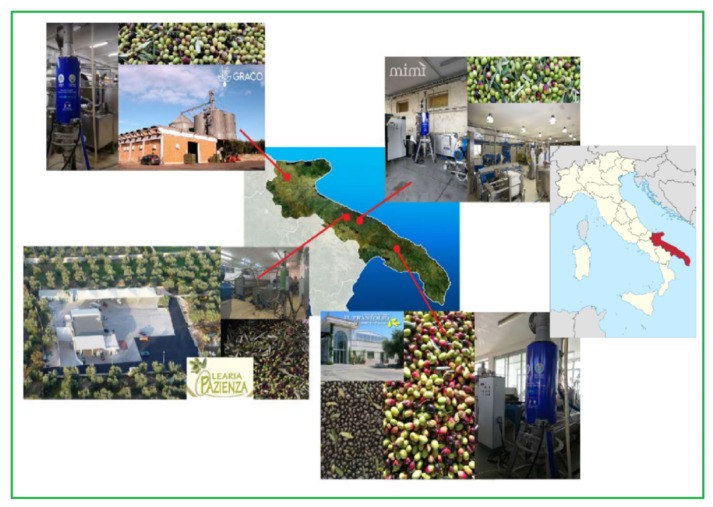
End-user validation of the industrial ultrasound application in the extra-virgin olive oil extraction process.

During the harvesting season in 2017, this economic support allowed for testing the innovative sono-heat exchanger by implementing the device in different processing lines, in different geographical areas, and with several olive cultivars (Figure 6).

The principle that led the experimentation was the total sharing of plant management with the olive millers. Test validation (TRL 7: Demonstration of system operating in an operational environment at a pre-commercial scale) is crucial to the successful development and technology readiness level (TRL) escalation of any new food technology.

Sharing the experimentation with the miller company allowed for gathering feedback useful in improving the technology and optimizing the results, which were still shared with the stakeholders, establishing a virtuous circle of trust and collaboration.

End-user validation (Figure 6) is a crucial process, as it allows the researcher not only to understand if the innovation meets the real needs of those who will be its users (the olive millers), but also to generate useful opinions for optimization directly from those who are insiders of a given sector. It is the best strategy to transform ultrasound technology into something that really meets the needs of the olive oil sector. It determines the desired radical change in the value of the product (healthier) and in the improvement of the income of the producers (more quantity of a higher-value product).

End-user validation of the industrial plant was really not so simple as it seems at first sight. The technical limits to be overcome in a real-scale experimentation were:

- The need to transport, mount, adapt (each plant limits and has different fittings that involve custom modifications), and test the machine by inserting it into different layouts for brands and types of devices upstream and downstream;
- The need to adapt the flow rates of the processing line to the best flow rate of the prototype;
- The need to have large homogeneous lots of olives (each test required a batch of 800 kg of olives); and

The need to coordinate research activity with the production needs of the olive millers. In fact, the experimental tests require to slow down the usual rate of processes due to the complex measurement operations, and the need to clearly separate the individual olive batches for each experimental test. Adding these difficulties in a period of frenetic activity, also conditioned by contracts with farmers that confer the olives to the oil mill, requires large efforts by the millers. The tests conducted for the validation of the technology showed that the innovative system, which combined the mechanical energy of ultrasound with the possibility of modulating the thermal exchange of the olive paste (heating or cooling), enabled the following:

- Eliminating malaxation by realizing an actually continuous process;
- Raising the extraction yields by recovering a further quota of extra-virgin olive oil that usually is lost in the pomace;
- Enhancing the antioxidant molecules that are lost in byproducts with traditional methods when the plant is set to raise oil yields;
- Obtaining a better organoleptic evaluation of the resulting virgin olive oil; and
- Offering a sustainable plant solution that can guarantee the right income to producers.

The close collaboration with the olive millers allowed an extremely technical approach to the system and highlighted the problems that had to be resolved to make the machine suitable for the market. At the end of the validation tests, each criticality was successfully resolved (TRL 8: Manufacturing issues solved).

In the aim to cover the last mile, there is a last step to climb to conclude the technology readiness level scale: TRL 9 (full commercial application, technology available for consumers). Among the European programs, such as Horizon 2020, the program “Fast Track to Innovation” (FTI), in particular, can support companies in assessing the potential feasibility and value of their idea and can provide information and advice on the best way forward and the needed financial support to reach TRL 9. In 2018, five partners from three European countries received from the European trough the program “Fast Track to Innovation” financial support to develop an ultrasound reactor, the solution for realize an effective continuous olive oil extraction process.The Fast Track to Innovation (FTI) program is a pilot project within the Horizon 2020 program that aims to support the market entry of innovative ideas, providing funding opportunities through a bottom-up approach. This means that the European Commission does not set out in detail the objectives of the call for proposals, but rather indicates the areas eligible for funding: The proposals submitted must indeed be linked to an area within the specific "leadership in enabling and industrial technologies "(KETs: Key enabling technologies) and/or one of the specific objectives of the" social challenges" pillar. FTI supports innovative projects from the demonstration phase to the commercialization of the product, including prototyping, testing, achievement of the necessary certifications, validation of business models, pre-normative research, and standardization. FTI is aimed at new market-oriented technologies, concepts, processes, and business models that need a last phase of development before they can be marketed (TRL 9).

Carlos Moedas (a Portuguese engineer, economist, and politician), the European Commissioner for Research, Science, and Innovation has said: “Europe has excellent science, but we lack disruptive market creating innovation. This is what is needed to turn our best ideas into new jobs, businesses, and opportunities". This is what is needed to turn our best ideas into new jobs, businesses, and opportunities”. Following this recommendation, five partners met together and, by means the cross-fertilization of ideas and competences, will contribute to accelerate the process of development of innovation. In just two years, they had to achieve the first marketable plant that combines ultrasound energy and heat exchange to overcome the current limits of the olive oil industry and bring to market a robust machine that is energy-efficient and capable of accelerating the extraction process by reducing the processing time, improving simultaneously and for the first time in the history of olive oil production plants the quantity and quality of the resulting oil. Olive sound project was proposed by a European partnership composed of five partners belonging to three countries.

The partnership consisted of the following:

- Two research institutions, the University of Bari supported by the Polytechnic of Bari (Italy) for the transfer of know-how gained in the development of technology (up to TRL 8); and Ctic Cita, for the validation of the pilot-scale technology (500 kg/h) (Spain);
- Two companies, Pieralisi, a leader in the market of oil machines (Italy), and Cedrat Technologies, a leader in the market of ultrasound devices (France); they designed and built the prototypes (500 and 4000 kg/h); and finally
- A large oil mill, Almazara, which tested the full-scale plant (4000 kg/h) for an entire olive growing campaign.

## 3. An Innovation That Looks to the 2030 Agenda for Sustainable Development

The 2030 Agenda for Sustainable Development is a plan of action for people, planet, and prosperity. It is articulated in 17 Sustainable Development Goals and 169 targets. Regarding the planet, the agenda aims to protect the planet from degradation, including through sustainable consumption and production, sustainably managing its natural resources and taking urgent action on climate change, so that it can support the needs of the present and future generations. The document designs the road toward a world in which consumption and production patterns and the use of all natural resources—from air to land, and from rivers, lakes, and aquifers to oceans and seas—are sustainable. Environmental protection requires the development and application of technology that is climate-sensitive, respects biodiversity, and is resilient in order to favor humanity living in harmony with nature. The document recommends making fundamental changes in the way that our societies produce and consume goods and services. In particular, Goal 2 (end hunger, achieve food security and improved nutrition, and promote sustainable agriculture) fixes this objective: By 2030, ensure sustainable food production systems and implement resilient agricultural practices that increase productivity and production and that help maintain ecosystems. Moreover, Goal 8 promotes the objective of improving progressively, through 2030, global resource efficiency in consumption and production and endeavoring to decouple economic growth from environmental degradation, in accordance with the 10-year framework of programs on sustainable consumption and production. Goal 12 fixes an objective that, by 2020, the environmentally sound management of chemicals and all wastes throughout their life cycles be achieved in accordance with agreed international frameworks, significantly reducing their release into the air, water, and soil in order to minimize their adverse impacts on human health and the environment.

Industrial ultrasound applications in the extra-virgin olive oil extraction process has taken into account these goals in order to develop an innovative time-saving and environmentally friendly process [43,44].

Ultrasound technology had already been used for more than 30 years by academia and industry, making preservation, transformation, and extraction greener. The design of green and sustainable processes is currently a hot research topic in the food industry, and ultrasound represents a multifaceted strategy able to interpret the concept of green food processing [42,43], helping to meet the challenges of the 21st century, protecting both the environment and consumers, and in the meantime enhancing competition in industries to be more ecological, economic, and innovative. In fact, it is well known that ultrasound can have a significant effect on the rate of various processes in the food industry, assuring fully reproducible food processes with high reproducibility, reducing the processing cost, simplifying manipulation and work-up, eliminating post-treatment of waste water, and consuming only a fraction of the time and energy normally needed for conventional processes [43].

The possibility of converting long malaxing times into a complete thermal and ultrasonic exchange that requires only a few minutes (or seconds depending on flow rates) has beneficial effects also on the amount of secondary metabolites (such as polyphenol and tocopherols characterized by heathy effects approved by the European Food Safety Authority) extracted from the olive paste. In fact, the long malaxing time required in the traditional olive oil extraction process represents a critical control point in the Nutrient Hazard Analysis and Critical Control Point Process (NACCP). Renzo et al., in 2015, developed a new procedure for the optimization of nutritional levels based on four general principles: (i) Guaranteeing health maintenance; (ii) evaluating and assuring the nutritional quality of food and total quality management; (iii) giving correct information to consumers; and (iv) ensuring an ethical profit. In light of this procedure, during malaxation a large number of technological parameters are beyond the control of the operator. In particular, it is difficult to have complete control of the factors (oxygen concentration, temperature, etc.) that affect the activation of desirable enzymatic reactions (synthesis of volatile compounds by the pathway of lipoxygenase) and the inhibition of undesired enzymatic reactions (oxidation of polyphenols by peroxidase and polyphenol oxidase) [3]. Considering the experimental evidence that showed that the innovative technology developed did not compromise the synthesis of aromas, and in light of the different enzymatic kinetics that see the pathway of lipoxygenase favored in terms of time compared to oxidative enzymes that in the process conditions require long activation times, it is possible to conclude that, contrary to what happens in other food industries, no degradation reaction could be attributed to the use of ultrasounds [31].

## 4. Key Questions on Industrial Ultrasound Applications in the Extra-Virgin Olive Oil Extraction Process

Recent scientific literature has offered a plethora of articles on ultrasound applications in the extra-virgin olive oil extraction process that have presented contradictory approaches to some technical choices to which answers must be given, supported by scientific evidence, in particular concerning the following:

- The frequency most suitable for effective cavitation;
- The specific energy necessary to achieve desired effects in a sustainable manner; and
- The opportunity to combine ultrasound with other emerging technologies.

The ultrasound frequency range extends from 20 kHz (low-frequency ultrasound) to almost 10 GHz (high-frequency ultrasound). The ultrasounds are produced by transducers that convert the energy obtained by applying a high potential difference in an ultrasonic wave, essentially a mechanical vibration, thanks to the piezoelectric effect of quartz, discovered by Curie in 1880. The phenomenon of cavitation, that is the creation of microscopic gaseous bubbles that implode, breaking the cell walls, can only take place in an aqueous medium below 200–300 kHz, since as the frequency increases, the attenuation induced by the inertia of the medium dampens the waves, nullifying the mechanical effect. The useful field of application to olive paste, which for density, viscosity, and structure (solid + two liquids immiscible among them) shows high attenuation actions on the wave able to dampen the vibrations induced by the transducer, is below 50 kHz. So-called megasounds (with frequencies of 400 and 600 kHz) are not able to induce the formation of cavities when traveling through a medium [45,46].

Sizing the power of an industrial plant is a function of the work capacity of the entire plant (the ultrasound device size has to be compatible with the capacity of the machines upstream and downstream), and it is a crucial element in obtaining a desired result in a sustainable manner, which depends on the specific energy transferred to the olive paste. Thus, the correct way to size the total power of the plant consists of an assessment of specific energy corresponding to the level of maximum effectiveness and efficiency, starting from a lab-scale experiment and reproducing the same conditions in a continuous plant.

In fact, by observing the curves correlating the time of sonication (fixed frequency and power) with the concentration of the minor compounds passing from olive paste to oil (Figure 2), it is possible to notice that as the sonication time increases, the quantity of extracted compounds increases. However, the curve presents first a linear section, followed by an elbow and a reduction in the slope of the curve. This graph clearly shows that within a certain interval of time, there is a direct correlation between the administered energy and the expected result. At a certain interval of time, at the elbow, when the administered energy is increased, the achievable result does not increase proportionally. Thus, the elbow represents the point of maximum extractive efficiency and energy efficiency.

Dividing the product between the time (*t*) and the power (*P*) for the mass of sonicated olive paste (*m*), it is possible to obtain the specific energy value (*Li*) given to the olive paste in a batch at the point of maximum efficiency and effectiveness. Knowledge of the specific energy value makes it possible to size the number of ultrasonic transducers and therefore the geometry (according to the flow rate (*G*) (kg/h) of the plant, which operates continuously), obtaining the same results by limiting the inputs:

(1) $$L_{i}=\frac{P*t}{m},$$

(2) $$P=\frac{G*L_{i}}{\eta_{us}},$$

The opportunity to combine ultrasound with other emerging technologies (e.g., microwave) is not consistent with some objectives toward which innovations tend:

- Constructive simplification and reduction of plant investment costs;
- Having extremely flexible heat exchange systems;
- Avoiding oxidative damage caused by so-called thermal spots induced by magnetron technology [47];
- Using non-energy-intensive technologies; and
- Limiting the technological actions that leave a chemical, nutritional, and organoleptic trace on the finished product (mild technologies and minimally processed foods).

Table 2 describes benefits and drawbacks of industrial ultrasound applications in the extra-virgin olive oil extraction process, summarizing the challenge that a researcher have to encounter to arrive to TRL 9, investing energy in multiple directions simultaneously, such as favoring the creation of a multidisciplinary research team, demonstrate ability to attract funding, and to disseminate results (Table 3).

## 5. The Role of Patents and Open Innovation in Accelerating Scientific Innovation

One of the rationales for patents is that they stimulate economic and technological development and promote competition by creating a financial motivation for invention in return for the disclosure of the invention to the public. Although the potential of the patent system has been widely recognized in the context of dynamic innovation activities, some critics have claimed that the current patent system stymies R&D and technological advances.

The scientist is an innovator par excellence. Innovation finds its foundation in the ability to create, find, combine, and transfer knowledge. Knowledge is the basis upon which the ability to innovate is based, and the university is the temple of knowledge.

Researchers working in public universities are called upon to make their skills and ingenuity available to solve problems that affect the territory in which the university operates to improve the living conditions and economic, social, and cultural well-being of its citizens.

Transferring an innovation in the world of extra-virgin olive oil is not a simple path, because one of the cornerstones of the product’s collective imagination is the concept of tradition rooted in the people’s minds.Furthermore, a huge obstacle to the validation of innovations is the seasonality of the product.

Starting from these premises, the researcher who develops innovative systems and who does not limit himself to testing machines developed by private companies to certify functional aspects finds himself at a crossroads when he makes a discovery:

- Patent the idea, the prototype, or the application by specifying that every result generated by the research activity with the means of the university is owned by the university itself, and the researcher cannot profit from it unless the results were obtained through development and patenting with personal resources (so the inventor is the researcher, but the patent is owned by the university); or
- Publish the results of the research (not disclosable throughout the patent investigation period), describing all of the design, construction, and experimental conditions in order to make the experiment reproducible by third parties (principle of reproducibility). This is the realization of an open intellectual property strategy and represents the path to foster a transition to more effective, efficient, and sustainable technologies.

Open innovation, or a free innovation sharing strategy, can therefore strengthen and catalyze development opportunities because it represents a way to facilitate the transition toward more effective, efficient, and sustainable technologies [48].

Fragmented intellectual property in a sector can slow down innovation and the adoption of a technology. In consideration of this, every single step in the development of the sono-heat-exchanger was published in detail without patenting ideas, models, and applications. The hope is that the availability of constructive data that make experiences perfectly replicable represents a technological accelerator capable of making large quantities of high-quality oil available to citizens on the market. This means more profit for the weakest players in the oil supply chain: The olive farmers and the olive millers.

## Figures and Tables

**Table 1:** Role of different disciplines in the trans-disciplinary approach to accelerate the development and implementation of industrial ultrasound applications in the extra-virgin olive oil extraction process.

Discipline	Role in the Transdisciplinary Approach
Food technologies	Generate new applications of emerging technologies in the different food supply chains. Test hypotheses on a laboratory scale, obtaining the parameters to be transferred to the mechanical engineers for plant design. Participate in testing and validation activities by studying the interaction between the emerging technology, the raw material, and its characteristics and effects on the chemical, sensory, and health characteristics of virgin olive oil, identifying the system to modulate the finished product from a quantitative and qualitative point of view [3].
Mechanical engineering	Invent numerical simulation models able to reproduce the mechanical and thermal effects of the emerging technology to develop innovative planning strategies able to convert science into product ready to the market (TRL from 1 to 9) [29,30,31]. Verify the effectiveness of the project by testing the machine in the real system. Develop strategy of scale-up. Evaluate the sustainability of the innovation.
Food chemistry	Evaluate the phenol content of the virgin olive oil and its antioxidant capacity [32]. Verify the possibility of enhancing the product value by means of the health claims approved by the European Food Safety Authority (EFSA) [33]. Study the effect of technological innovation on the shelf life of the product.
Medicine	Develop in vitro and in vivo experimental models to discover new beneficial effects of virgin olive oil extract by means of emerging technologies [34].
Food marketing	Study factors determining neophobia and neophilia with regard to emerging technologies applied to the olive oil sector. Measure consumers’ willingness to pay for virgin olive oil extracted by means of emerging technologies [35].
Agri-food law	Verify the compatibility between technological innovations based on emerging technologies and the legislation in force on product classification of olive oils. Encourage the diffusion of technological innovations based on emerging technologies by developing legislative proposals that encourage the production of healthy olive oil (certified by claims) through incentives based on tax relief for olive millers and tax exemptions for consumers.
Sensory science	Study the effect of technological innovations based on emerging technologies on the sensory characteristics of the product [31].

A transdisciplinary approach is characterized by its focus on “wicked problems” that need creative solutions, a reliance on stakeholder involvement, and engaged socially responsible science. In simultaneously studying multiple levels and angles of reality, transdisciplinary work provides an intriguing potential to invigorate scientific inquiry both in and outside the academy [25].

**Table 2:** Benefits and drawbacks of industrial ultrasound applications in the extra-virgin olive oil extraction process.

Advantages	Disadvantages
Ultrasound is a cost-effective and efficient alternative compared to traditional extraction techniques. Ultrasound increases the oil yield and acceleratesfavorable enzymatic kinetics with respect to traditional extraction techniques. Ultrasound facilitates the extraction of healthy minor compounds. The costs of equipment are lower than those of other emerging technologies. Ultrasound enhances extraction efficiency and extraction rate, with moderate increments of temperature. Ultrasound was found to have no detrimental effect on the composition of oil, and on the contrary improved antioxidant content.	Wave attenuation in the olive paste and a decrease in the sound wave amplitude with distance are major challenges in the development of an ultrasound device. The activated ultrasound zone is restricted to a limited zone in the vicinity of the ultrasound emitter. There is no commercial software useful in predicting the activated ultrasound zone. The necessity to perform a simulation of pressure transients induced by the sound wave through the olive paste with fluid dynamic phenomena implies to spend long-time for the complex numerical study olive pasteThe result of the numerical study changes every time the work capacity of the extraction plant changes.

**Table 3:** Mix of skills needed for a rapid TRL ascent: Multidisciplinary skills of the research team, ability to attract funding, and dissemination of results.

TRL Level	Description	Papers	Sources of Funding
**TRL 0:** **Unproven concept, no testing has been performed**	The first step of this research was represented by an overview of the potential application of emerging technologies in the virgin olive oil extraction process. During this evaluation, the choice of strategies useful in developing innovative plants represented the transition from TRL 0 to TRL 2. Starting from the development of the idea (TRL 0) and examining the principles postulated and observed without any experimental proof available (TRL 1), the potential of a group of emerging technologies (ultrasound, microwaves, and pulsed electric fields) was analyzed in order to improve the virgin olive oil extraction process. The end part of these first steps was concluded with the formulation of a hypothesis of the application of ultrasound technology to the virgin olive oil extraction process (TRL 2).	[3,6,8,13,36]	University of Bari funds
**TRL 1:** **Basic research**			
**TRL 2:** **Technology formulation**			
**TRL 3:** **Applied research, proof of concept**	The first laboratory tests (TRL 3 and TRL 4) were conducted in 2012, employing a micro-olive mill with a work capacity equal to 2.5 kg/h combined with an ultrasound bath.	[17,25]	EU through the Molise Region; European Agricultural Fund for Rural Development; Europe invested in rural areas under Measure 124 (second edition) of PSR Molise 2007/2013 Determination of concession no. 108
**TRL 4:** **Small-scale prototype built in a laboratory environment**			
**TRL 5:** **Large-scale prototype tested in intended environment**	The first machine composed of a tube-in-tube heat-exchanger combined with an ultrasound probe that allowed the application of ultrasound technology at a full-scale industrial level represented TRL 5.	[31]	
**TRL 6:** **Technology demonstrated in relevant environment**	During the harvesting season in 2016, a new prototype system was tested in an industrial olive mill, transforming tens of tons of olives in oil of high quality and rich in polyphenols, with a concentration compatible with an application of the health claim of biophenols approved by the European Food Safety Authority (EFSA) and with the expected performance (TLR 6).	[19,29,38]	Regional program to support smart specialization, social sustainability, and environmental intervention: “FUTURE IN RESEARCH” (€ 150,000.00), three years Of a Temporary researcher- RTD-A payed to work on the subject “Ultrasound applied to of the virgin olive oil extraction process”. EU through the Apulia Region: Support to the Regional Technological Innovation Clusters Project: “PERFORM TECH (Apulian EMERGING FOOD TECHNOLOGY), food safety through the use of emerging technologies in the development of functional products, recovery of nutraceutical substances from byproducts, and enhancement of energy waste”, code LPIJ9P2 AGER 2 Project, grant in 2016: 0174 AGER FOUNDATION , Sector: olive tree and olive oil – Title: COMPETiTiVE: Claims of olive oil to improve the market value of the product
**TRL 7:** **System prototype, demonstration in operational environment**	During the harvesting season in 2017, a new economic support allowed for the testing of the innovative sono-heat exchanger by implementing the device in different processing lines, in different geographical areas, and with several olive cultivars. This is the “end user validation” of an industrial ultrasound application in the extra-virgin olive oil extraction process. The principle that led the experimentation was the total sharing of plant management with the olive millers. Test validation (TRL 7) is crucial to the successful development and technology readiness level escalation of any new food technology. Sharing the experimentation with the miller company allowed for gathering feedback useful in improving the technology and optimizing the results, which were still shared with the stakeholders, establishing a virtuous circle of trust and collaboration. The close collaboration with the olive millers allowed an extremely technical approach to the system and highlighted the problems that had to be resolved to make the machine suitable for the market. At the end of the validation tests, each criticality was successfully resolved (TRL 8).		
**TRL 8:** **System complete and qualified**			
**TRL 9:** **Actual system proven in operational environment**	This is a work in progress (2019–2021) toward a product ready for the market.		EU project 820587—OLIVE-SOUND—ultrasound reactor: The solution for a continuous olive oil extraction process; H2020-EU.2.1.—INDUSTRIAL LEADERSHIP-EIC-FTI- 2018–2020—Fast Track to Innovation (FTI)
